# The effect of tobacco smoking and treatment strategy on the one-year mortality of patients with acute non-ST-segment elevation myocardial infarction

**DOI:** 10.1186/1471-2261-10-59

**Published:** 2010-12-15

**Authors:** Erlend Aune, Knut Endresen, Jo Roislien, Joran Hjelmesaeth, Jan Erik Otterstad

**Affiliations:** 1 Department of Cardiology, Vestfold Hospital Trust, Toensberg, Norway; 2 Department of Cardiology, Rikshospitalet University Hospital, Oslo, Norway; 3 Department of Biostatistics, Institute of Basic Medical Sciences, University of Oslo, Oslo, Norway; 4 Morbid Obesity Center, Vestfold Hospital Trust, Toensberg, Norway

## Abstract

**Background:**

The aim of the present study was to investigate whether a previously shown survival benefit resulting from routine early invasive management of unselected patients with acute non-ST-segment elevation myocardial infarction (NSTEMI) may differ according to smoking status and age.

**Methods:**

Post-hoc analysis of a prospective observational cohort study of consecutive patients admitted for NSTEMI in 2003 (conservative strategy cohort [CS]; n = 185) and 2006 (invasive strategy cohort [IS]; n = 200). A strategy for transfer to a high-volume invasive center and routine early invasive management was implemented in 2005. Patients were subdivided into current smokers and non-smokers (including ex-smokers) on admission.

**Results:**

The one-year mortality rate of smokers was reduced from 37% in the CS to 6% in the IS (p < 0.001), and from 30% to 23% for non-smokers (p = 0.18). Non-smokers were considerably older than smokers (median age 80 vs. 63 years, p < 0.001). The percentage of smokers who underwent revascularization (angioplasty or coronary artery bypass grafting) within 7 days increased from 9% in the CS to 53% in the IS (p < 0.001). The corresponding numbers for non-smokers were 5% and 27% (p < 0.001). There was no interaction between strategy and age (p = 0.25), as opposed to a significant interaction between strategy and smoking status (p = 0.024). Current smoking was an independent predictor of one-year mortality (hazard ratio 2.61, 95% confidence interval 1.43-4.79, p = 0.002).

**Conclusions:**

The treatment effect of an early invasive strategy in unselected patients with NSTEMI was more pronounced among smokers than non-smokers. The benefit for smokers was not entirely explained by differences in baseline confounders, such as their younger age.

## Background

Early invasive management of non-ST-segment elevation myocardial infarction (NSTEMI) has been shown, when contrasted with a conservative treatment approach, to improve clinical outcome [1]. Whether such an effect differs between smokers and non-smokers is difficult to explore, since smokers with NSTEMI are substantially younger than non-smokers. To the best of our knowledge, such an exploration has only been attempted in a sub-analysis of the FRISC II study [2], where allocation to early invasive treatment for non-ST-segment elevation acute coronary syndrome (NSTE-ACS) was associated with a clinical benefit in non-smokers only [3]. The risk level was moderate, as reflected in a 6 months mortality rate of 2%. The favorable effect of early invasive management was exclusively driven by a reduction of recurrent MI and no information on age-differences was provided. In a study of unselected patients with acute myocardial infarction (AMI), we found that for NSTEMI the one-year mortality rates were 32% with conservative management and 21% with an early invasive approach [4]. The purpose of the present analysis was to investigate whether this survival benefit may differ according to smoking status and age.

## Methods

Details on the study design and methodology are published elsewhere [4]. In brief, all patients referred to our non-invasive hospital during two one-year periods with a suspected AMI were prospectively registered. The diagnosis of AMI was made in accordance with the European Society of Cardiology/American College of Cardiology criteria of 2000 [5]. The conservative strategy cohort (CS) included patients admitted from February 1, 2003 through to January 31, 2004. The invasive strategy cohort (IS) included patients admitted from February 15, 2006 through to February 14, 2007. Patients were transferred approximately 100 km (63 miles) to the closest high-volume invasive center (Rikshospitalet University Hospital, Oslo, Norway).

A diagnosis of AMI was, in the case of both cohorts, made in the presence of typical symptoms and elevated troponin T greater than a cutoff level of ≥ 0.1 μg/L. According to the electrocardiographic findings, AMI was sub-classified into ST-segment elevation myocardial infarction (STEMI) and NSTEMI. Current smokers included those who had smoked within the last three months. Non-smokers were defined as never-smokers and ex-smokers who had stopped more than three months prior to admission. Data on smoking cessation was not collected due to the fact that informed consent was required in order to undergo one-year follow-up on morbidity and medication. This, however, could only be obtained in 55% of the study group due to limitations such as patient refusal, old age, dementia and geographical factors. Baseline risk evaluation for both cohorts included the Global Registry of Acute Coronary Events (GRACE) risk score for 6-month mortality [6]. The primary outcome was all-cause death after one year.

Patients with NSTEMI have platelet-rich thrombi, as opposed to predominantly fibrin-rich thrombi in STEMI [7]. We therefore felt it appropriate to evaluate separately the impact of tobacco smoking for each type of AMI. The present report addresses NSTEMI patients only, since it would be underpowered to explore the impact of smoking status in the smaller subset of STEMI patients.

Both the regional ethics committee for South-East Norway Regional Health Authority and the Norwegian Social Science Data Services approved the study.

### Statistical analysis

In the post-hoc analysis, Mann-Whitney U test was used for comparison of continuous data between different groups of patients. Proportions were analyzed by χ^2 ^test or Fisher's exact test. Kaplan-Meier plots and Log rank tests were used for unadjusted comparison of survival between different subsets of patients, i.e. smokers and cohort. Two multiple Cox proportional hazards regression models were used for additional survival analyses. In model 1, explanatory variables with a p-value ≤ 0.05 in the main study's multiple regression analysis [4] (treatment strategy, age, s-creatinine and previous left ventricular systolic dysfunction), in addition to smoking status at admission, as well as aspirin and statin usage during hospitalization, were used to assess the hazard ratio (HR) for death after one year. Interaction terms between age/strategy and smoking/strategy were included and tested. In model 2, GRACE risk score (including age, heart rate, systolic blood pressure, s-creatinine, Killip Class, cardiac arrest at admission, ST-segment deviation and elevated cardiac markers) was used for the adjustment of differences at baseline risk, with the analysis presented separately for smokers and non-smokers. In both models, the cohort was used as a surrogate variable for the treatment strategy. The assumption of proportional hazards was explored with partial residual plots. Two-tailed p-values below 0.05 were considered statistically significant. The analyses were implemented using SPSS^® ^16.0 (SPSS Inc, Chicago, IL).

## Results

In 2003 (CS) 185 patients were admitted with NSTEMI. Data on smoking status was obtained in 181 cases (98%), of whom 54 (30%) were current smokers. In 2006 (IS) 200 patients were admitted with NSTEMI. Data on smoking status was complete, with 49 (25%) patients found to be current smokers (p = 0.29 versus CS). Baseline characteristics according to smoking status at admission and treatment cohort are presented in Table 1. Smokers were significantly younger than non-smokers both in the IS (median age 60 vs. 81 years, p < 0.001) and the CS (median age 66 vs. 79 years, p < 0.001). Smokers in the IS had a significantly lower s-creatinine (median [25^th^-75^th ^percentile] 76 [69-96] vs. 95 [75-113] μmol/L, p = 0.014) compared with smokers in the CS. More smokers in the IS had prior PCI when compared to smokers in the CS. Otherwise, baseline risk factors, including GRACE risk score, were similar among smokers and non-smokers within both cohorts.

**Table 1:** Baseline characteristics for NSTEMI patients.

	Non-smokers			Smokers*		
		
	CS (n = 127)	IS (n = 151)	p-value	CS (n = 54)	IS (n = 49)	p-value
Age (years)	79 (72-86)	81 (69-86)	0.61	66 (56-76)	60 (55-72)	0.17
Male	74 (58%)	87 (58%)	1.00	39 (72%)	34 (69%)	0.92
*Medical history*						
Diabetes	21 (17%)	29 (19%)	0.67	6 (11%)	6 (12%)	1.00
Previous AMI	42 (33%)	53 (35%)	0.82	14 (26%)	12 (25%)	1.00
Previous LVSD†	10 (8%)	19 (13%)	0.30	7 (13%)	3 (6%)	0.40
Hypertension	46 (36%)	45 (30%)	0.31	17 (32%)	13 (27%)	0.74
Stroke	6 (5%)	17 (11%)	0.080	6 (11%)	2 (4%)	0.34
CABG	11 (9%)	20 (13%)	0.31	5 (9%)	3 (6%)	0.82
PCI	6 (5%)	10 (7%)	0.68	3 (6%)	10 (20%)	0.049
*Presenting characteristics*						
S-Creatinine, μmol/L‡	95 (76-128)	90 (77-115)	0.30	95 (75-116)	76 (69-97)	0.014
GRACE risk score	140 (113-166)	139 (110-164)	0.53	112 (84-160)	108 (80-131)	0.28

Categorical data presented as n (%) and continuous data as median (25^th ^-75^th ^percentile). *Smoking within last three months. †Defined as prior left ventricular ejection fraction < 40%. ‡Conversion factor 0.0113 for mg/dL.

AMI, acute myocardial infarction; CABG, coronary artery bypass grafting; CS, conservative strategy cohort; GRACE, global registry of acute coronary events; IS, invasive strategy cohort; LVSD, left ventricular systolic dysfunction; NSTEMI, non-ST-segment elevation myocardial infarction; PCI, percutaneous coronary intervention; STEMI, ST-segment elevation myocardial infarction.

### Total mortality

**Figure 1:**
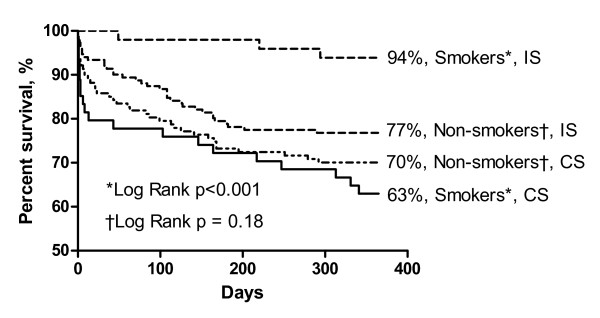
**One-year survival in non-ST-segment elevation myocardial infarction (NSTEMI) patients with invasive strategy (IS) and conservative strategy (CS)**.

The Kaplan-Meier estimates of one-year survival according to smoking status and treatment strategy are shown in Figure 1. Smokers in the CS had a one-year mortality of 37% (20/54) as compared with 6% (3/49) in the IS (p < 0.001). The corresponding numbers for non-smokers were 30% (38/127) and 23% (35/151) (p = 0.18).

The results from the Cox proportional hazards regression analyses are presented in Table 2 (model 1) and Table 3 (model 2). In model 1, a statistically significant interaction was found between strategy and smoking (p = 0.024). Current smoking was an independent predictor of mortality (HR 2.61, 95% confidence interval [CI] 1.43-4.79, p = 0.002). No interaction was observed between strategy and age (p = 0.25). When adjusted for GRACE risk score at admission (model 2) IS was associated with a statistically significant reduction of one-year mortality for smokers (HR 0.20, 95% CI 0.06-0.68, p = 0.010), but not for non-smokers (HR 0.79, 95% CI 0.49-1.28, p = 0.34).

**Table 2:** Hazard ratios (HR) of death in patients with NSTEMI (n = 381) during one-year follow-up using multiple Cox proportional hazards regression (Model 1).

	HR	95% CI	p-value
Invasive strategy	0.80	0.50-1.27	0.34
Age per year	1.05	1.02-1.08	< 0.001
S-creatinine per unit (μmol/L)	1.005	1.003-1.007	< 0.001
Current smoking	2.61	1.43-4.79	0.002
Previous LVSD*	1.63	0.97-2.75	0.064
Statin during hospitalization	0.46	0.29-0.71	0.001
Aspirin during hospitalization	0.57	0.35-0.90	0.017
Interaction term (current smoker/strategy)	0.22	0.06-0.82	0.024

*Defined as previous left ventricular ejection fraction < 40%.

CI, confidence interval; LVSD, left ventricular systolic dysfunction; NSTEMI, non-ST-segment elevation myocardial infarction.

**Table 3:** Hazard ratios (HR) of death in patients with NSTEMI (n = 381) during one-year follow-up using multiple Cox proportional hazards regression according to smoking status (Model 2).

	Non-smokers (n = 278)			Smokers (n = 103)		
		
	HR	95% CI	p-value	HR	95% CI	p-value
Invasive strategy	0.79	0.49-1.28	0.34	0.20	0.06-0.68	0.010
GRACE risk score (per point)	1.03	1.02-1.04	< 0.001	1.04	1.02-1.05	< 0.001

CI, confidence interval; GRACE, global registry of acute coronary events; NSTEMI, non-ST-segment elevation myocardial infarction.

### Invasive procedures and mortality within 7 days

**Figure 2:**
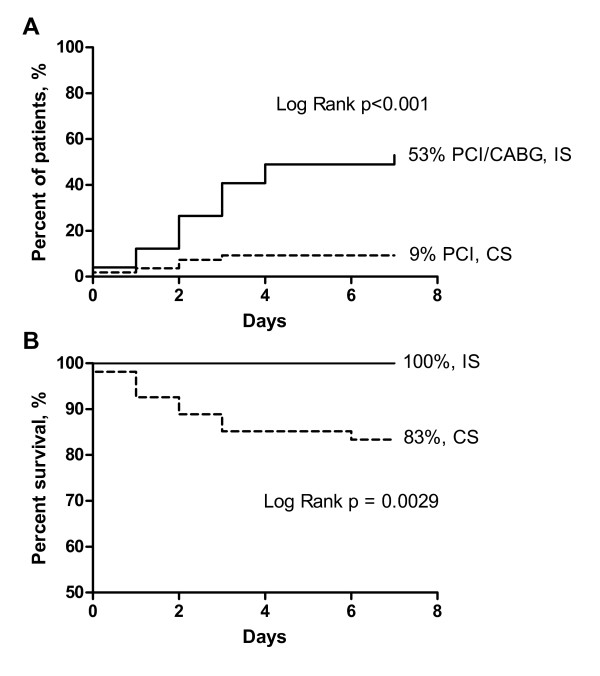
**Revascularization (a) and survival (b) during first 7 days in smokers with non-ST-segment elevation myocardial infarction (NSTEMI)**.

Among smokers, the proportion of patients who underwent coronary angiography within 7 days increased from 11% in the CS to 78% in the IS. This was accompanied by a 6-fold increase in percutaneous coronary intervention (PCI) and coronary artery bypass grafting (CABG) during the same time period (Figure 2a). The 7-days mortality rate reduced from 17% in the CS to 0% in the IS (Figure 2b).

**Figure 3:**
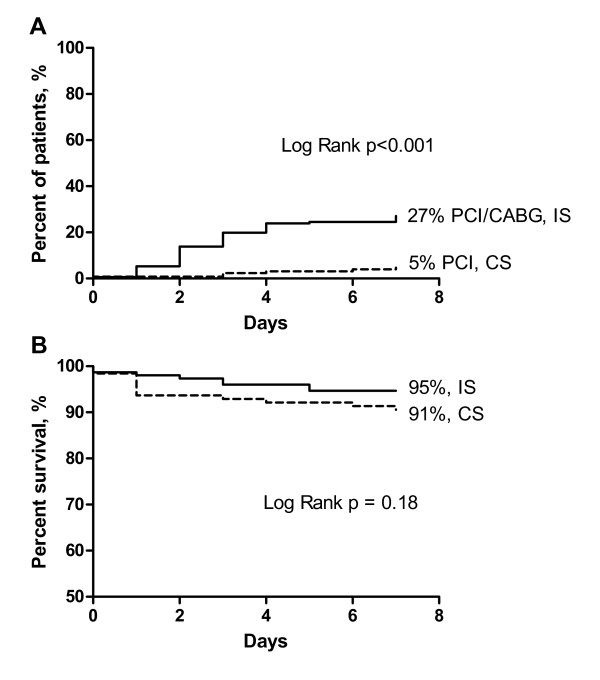
**Revascularization (a) and survival (b) during first 7 days in non-smokers with non-ST-segment elevation myocardial infarction (NSTEMI)**.

The proportion of non-smokers who underwent coronary angiography within 7 days was 6% in the CS and 49% in the IS. The corresponding data for early revascularization and death are presented in Figure 3a and Figure 3b, indicating a less pronounced and non-significant decrease in mortality as compared with smokers.

Non-smokers in the IS who had revascularization within 7 days were significantly younger than those not undergoing this treatment (median [25^th^-75^th ^percentile] age 71 [62-77] vs. 83 [74-88] years, p < 0.001). The corresponding figures for smokers were 58 (50-70) vs. 63 (59-78) years (p = 0.035). Similar age-differences were observed in the CS.

Among patients treated with PCI, 95% had stents. There was no difference in the usage of drug-eluting stents (DES) in the IS between smokers and non-smokers (24% vs. 21%, respectively, p = 1.00).

### Medical treatment

Data on medical treatment during hospitalization are provided in Table 4. A non-significant tendency for more statin usage in the IS and for more aspirin usage among smokers than non-smokers was observed. Otherwise, the medication prescribed was similar within the two treatment cohorts, both for smokers and non-smokers.

**Table 4:** Medical treatment during index hospitalization in NSTEMI patients.

	Non-smokers			Smokers		
		
	CS (n = 127)	IS (n = 151)	p-value	CS (n = 54)	IS (n = 49)	p-value
Aspirin	103 (81%)	124 (82%)	0.95	48 (90%)	45 (92%)	0.86
Clopidogrel	92 (72%)	118 (78%)	0.34	46 (85%)	46 (94%)	0.27
Beta-blocker	110 (87%)	123 (82%)	0.32	46 (85%)	42 (86%)	1.00
ACE-I/ARB	53 (42%)	77 (51%)	0.16	22 (41%)	19 (39%)	1.00
Statins	74 (58%)	106 (70%)	0.051	40 (74%)	44 (90%)	0.072

Categorical data presented as n (%).

ACE-I, angiotensin converting enzyme inhibitor; ARB, angiotensin-II receptor blocker; CS, conservative strategy cohort; IS, invasive strategy cohort; NSTEMI, non-ST-segment elevation myocardial infarction.

## Discussion

### Principal findings

The main finding of this study is that smokers with NSTEMI benefit the most from an early invasive treatment strategy, and that the treatment effect seems independent of age. For all-cause mortality the interaction term between current smoker and strategy was significant, implying that the effect of an invasive strategy was significantly different between smokers and non-smokers. In this unselected population one-year mortality for smokers decreased from 37% in the CS to 6% in the IS. Half of the fatal events among smokers in the CS occurred within 7 days. Such a profound reduction in early mortality could not be demonstrated among the non-smokers, where the increased use of early invasive management was less pronounced. In spite of the favorable findings among smokers undergoing early invasive treatment, current smoking was still an independent predictor of one-year mortality.

### Comparisons with previous studies

Several studies and registries of patients with NSTE-ACS have compared the outcome of smokers vs. non-smokers in order to evaluate the possible existence of a "smoker's paradox" [1,8-10]. The consensus has been that the apparently favorable outcome among younger smokers is eliminated when adjustments for baseline risk factors are made in various multiple regression analyses. To the best of our knowledge, the only study that has compared the influence of early invasive vs. a conservative approach in smokers vs. non-smokers with NSTE-ACS is the FRISC II trial [2]. In contrast with our study, a clinical benefit resulting from early invasive treatment was observed for non-smokers but not for smokers. It must be emphasized that the study populations were different, with a much higher mortality rate in our observational study of unselected NSTEMI patients. In FRISC II only 68% of the patients had elevated troponin and age > 75 years was an exclusion criterion. In our study, 53% of the patients were > = 75 years old. Patients treated with PCI in FRISC II were unable to be treated with DES and only 61% had stents in the invasive group, whilst anti-platelet therapy comprised of ticlopidine treatment for only 3-4 weeks after the procedure. This is in contrast to our study, where the majority had stents, and all had clopidogrel prior to and 9 months after the procedure. In FRISC II, early invasive vs. conservative treatment had no influence on total mortality. As is reflected in the favorable influence on mortality in our study, it seems fair to assume that the introduction of early invasive management may have induced a reduction in both cardiac morbidity and total mortality.

Most studies on the prognostic impact of smoking in acute coronary syndromes have compared younger smokers with older non-smokers, and, in part, ex-smokers [8,9,11-13]. It may be speculated that smokers and non-smokers of similar age suffering from an AMI would differ in terms of other risk factors as well as the composition of the atherosclerotic lesion rendering them open to different treatment results.

### Differences in baseline confounding factors

Smokers were considerably younger and more likely to undergo early revascularization than non-smokers. It could therefore be argued that the favorable effect of an invasive strategy in the younger subgroup of smokers can be explained solely by this difference in age and the lower proportion of early revascularization in non-smokers. However, we found no interaction between strategy and age, as opposed to a significant interaction between strategy and smoking status at admission. Accordingly, the survival benefit of an invasive strategy in our study seems independent of age. This is consistent with previous findings from randomized trials. In the RITA 3 trial [14] patients assigned an early intervention for NSTE-ACS had a more favorable outcome than those with a conservative strategy, with the results consistent across various subgroups, including age. In two other large randomized studies [2,15] exploring the effect of early invasive strategy versus a conservative approach in NSTE-ACS there were no differences in outcome among patients subdivided into age groups ≥ 65 years and < 65 years. Based upon these considerations, it seems unlikely that differences in age and the proportion of smokers and non-smokers treated with early invasive strategy can solely explain the favorable results obtained among smokers undergoing early invasive versus conservative management.

Another potential confounding factor is the different concomitant medical therapy given to each cohort. It should be emphasized that there were no changes in the recommendations for adjunctive medical treatment from the first to the second cohort. In spite of this, we observed a non-significant tendency for more statin usage among smokers in the IS than in the CS. Statin and aspirin use during hospitalization were associated with a lower hazard ratio for mortality (model 1), but when included in the adjusted analysis, such treatment had no influence on the interaction between smoking and treatment strategy.

### Strengths and limitations of the study

The present study is a post-hoc analysis of a prospective observational cohort study including all patients with NSTEMI admitted to our hospital. This unselected study population is representative of Norwegian patients with NSTEMI in general, as opposed to patients included in randomized trials with various inclusion and exclusion criteria. On the other hand, due to the observational and nonrandomized design, our findings may have been influenced by unidentified confounders. In light of the relatively modest population size, potential differences between the groups might not be statistically significant because of type 2 errors. Due to the same reason it did not seem appropriate to stratify age groups. Data on important confounding factors during follow-up, such as secondary medical prophylaxis and smoking cessation after discharge were not available. According to observations made in studies of smoking cessation in patients with coronary heart disease, an apparent affect on mortality is not seen until after 2-4 years [16-18]. In recently published data from the OASIS 5 study of NSTE-ACS patients, smoking cessation was associated with a lower rate of reinfarctions but had no effect on mortality after 6 months [19]. The early mortality reduction observed in our study can therefore not be explained by a higher percentage of quitters in the IS than in the CS. Although smokers in the IS were slightly younger, had significantly lower s-creatinine and more statin treatment during the index hospitalization, the favorable mortality results were still statistically significant after adjustment for these confounders. Due to regulatory limitations we were not able to study the influence on recurrent myocardial infarctions. Finally, non-smokers were due to higher age and more co-morbidity less likely to undergo invasive treatment. This could have influenced our results in some way not accounted for.

## Conclusions

Unselected smokers with NSTEMI represent a subset of patients who receive particular clinical benefit from an early invasive strategy. This benefit seems to be independent of age.

## Abbreviations

ACE-I: angiotensin converting enzyme inhibitor; AMI: acute myocardial infarction; ARB: angiotensin-II receptor blocker; CABG: coronary artery bypass grafting; CI: confidence interval; CS: conservative strategy cohort; DES: drug-eluting stent; GRACE: global registry of acute coronary events; HR: hazard ratio; IS: invasive strategy cohort; LVSD: left ventricular systolic dysfunction; NSTE-ACS: non-ST-segment elevation acute coronary syndrome; NSTEMI: non-ST-segment elevation myocardial infarction; PCI: percutaneous coronary intervention; STEMI: ST-segment elevation myocardial infarction.

## Competing interests

The authors declare that they have no competing interests.

## Authors' contributions

This study was conceived and designed by EA, KE, JJ and JEO. EA, KE and JEO acquired the data. EA, KE, JR, JJ and JEO analyzed and interpreted the data. EA and JR conducted the statistical analysis. EA, JJ and JEO drafted the original version of the manuscript. EA, KE, JR, JJ and JEO revised the manuscript critically for important intellectual content. All authors have read and approved the final manuscript.

## Pre-publication history

The pre-publication history for this paper can be accessed here:

http://www.biomedcentral.com/1471-2261/10/59/prepub
